# In-Bed Sensorimotor Rehabilitation in Early and Late Subacute Stroke Using a Wearable Elbow Robot: A Pilot Study

**DOI:** 10.3389/fnhum.2021.669059

**Published:** 2021-05-24

**Authors:** Mei Zhen Huang, Yong-Soon Yoon, Jisu Yang, Chung-Yong Yang, Li-Qun Zhang

**Affiliations:** ^1^Department of Physical Therapy and Rehabilitation Science, School of Medicine, University of Maryland, Baltimore, MD, United States; ^2^Department of Rehabilitation Medicine, Presbyterian Medical Center, Jeonbuk, South Korea; ^3^Department of Neuroscience and Behavioral Biology, College of Arts and Sciences, Emory University, Atlanta, GA, United States; ^4^Department of Physical Medicine and Rehabilitation, The Seum Hospital, Jeonbuk, South Korea; ^5^Department of Orthopaedics, University of Maryland, Baltimore, MD, United States; ^6^Department of Bioengineering, University of Maryland, College Park, MD, United States

**Keywords:** stroke rehabilitation, robot, recovery time course, upper limbs, subacute stroke

## Abstract

**Objects**: To evaluate the feasibility and effectiveness of in-bed wearable elbow robot training for motor recovery in patients with early and late subacute stroke.

**Methods**: Eleven in-patient stroke survivors (male/female: 7/4, age: 50.7 ± 10.6 years, post-stroke duration: 2.6 ± 1.9 months) received 15 sessions of training over about 4 weeks of hospital stay. During each hourly training, participants received passive stretching and active movement training with motivating games using a wearable elbow rehabilitation robot. Isometric maximum muscle strength (MVC) of elbow flexors and extensors was evaluated using the robot at the beginning and end of each training session. Clinical measures including Fugl-Meyer Assessment of upper extremity (FMA-UE), Motricity Index (MI) for upper extremities, Modified Ashworth Scale (MAS) were measured at baseline, after the 4-week training program, and at a 1-month follow-up. The muscle strength recovery curve over the training period was characterized as a logarithmic learning curve with three parameters (i.e., initial muscle strength, rate of improvement, and number of the training session).

**Results**: At the baseline, participants had moderate to severe upper limb motor impairment {FMA-UE [median (interquartile range)]: 28 (18–45)} and mild spasticity in elbow flexors {MAS [median (interquartile range)]: 0 (0–1)}. After about 4 weeks of training, significant improvements were observed in FMA-UE (*p* = 0.003) and MI (*p* = 0.005), and the improvements were sustained at the follow-up. The elbow flexors MVC significantly increased by 1.93 Nm (95% CI: 0.93 to 2.93 Nm, *p* = 0.017) and the elbow extensor MVC increased by 0.68 Nm (95% CI: 0.05 to 1.98 Nm, *p* = 0.036). Muscle strength recovery curve showed that patients with severe upper limb motor impairment had a greater improvement rate in elbow flexor strength than those with moderate motor impairment.

**Conclusion**: In-bed wearable elbow robotic rehabilitation is feasible and effective in improving biomechanical and clinical outcomes for early and late subacute stroke in-patients. Results from the pilot study suggested that patients with severe upper limb motor impairment may benefit more from the robot training compared to those with moderate impairment.

## Introduction

Stroke is the leading cause of long-term disability among adults in the United States (Virani et al., 2020) and worldwide (Johnson et al., 2016). More than 795,000 people suffer a stroke in the United States each year (Virani et al., 2020), and nearly three-quarters of all strokes occur in people over the age of 65 (Virani et al., 2020). With an ever-increasing elderly population, the stroke will continue to be a major health issue (Virani et al., 2020). Up to 70% of stroke survivors have hemiparesis affecting the upper extremity and about two-thirds of the stroke survivors demonstrate a long-term reduction in upper limb motor function (Kwakkel et al., 2003; Lee et al., 2015), which restrict their ability to perform everyday activities, reduce productivity, and limit social activities (Buma et al., 2013; Lee et al., 2015; Johnson et al., 2016; Virani et al., 2020). Improving upper limb function is a core element of stroke rehabilitation needed to maximize patient outcomes and reduce disability.

The first few months post-stroke are critical for motor recovery (O’dwyer et al., 1996; Kwakkel et al., 2003; Krakauer, 2006; Mirbagheri et al., 2009; Lee et al., 2015; Winstein et al., 2016; Kundert et al., 2019), when neural circuits reorganization, including spontaneous recovery and learning–dependent processes, dominate during the acute and subacute stages (Kwakkel et al., 2003; Krakauer, 2006; Lee et al., 2015). However, multiple studies worldwide have shown that for hospitalized stroke patients, 50–70% of the daytime they were inactive in their ward (Bernhardt et al., 2004; Lang et al., 2007; West and Bernhardt, 2012; Luker et al., 2015), and the time to receive physical therapy and occupational therapy was estimated to be less than 3 h per day (Bernhardt et al., 2004; West and Bernhardt, 2012). The duration of the therapeutic session was about 30 min, while the repetition for passive and active movement in the upper limb was about 33–50 (Lang et al., 2009). Moreover, observation showed that affected upper extremity use is minimal (3.3 ± 1.8 h) during the inpatient rehabilitation stay (Lang et al., 2007). Patients with severe motor impairment may have few engagements in the physical activity and intervention for the affected limb (Luker et al., 2015). However, it has been widely recognized that the effective way to promote neuroplasticity and functional motor recovery poststroke is intensive treatments (Buma et al., 2013) through specific functional (Van Peppen et al., 2004) and repetitive motor tasks (French et al., 2016). Apparently, most of the current inpatient stroke rehabilitation interventions cannot provide the desired training.

Over the past two decades, rehabilitation robots, with the capability to increase the number of movement repetitions in a given time compared to conventional therapy and provide individualized foundational tasks without requiring constant therapist involvement, have gained much attention in stroke rehabilitation (Volpe et al., 2000; Veerbeek et al., 2017). Moreover, robotic devices may also provide a timely quantitative and sensitive evaluation of the biomechanical performance of the patients (Ren et al., 2017), which can aid clinicians to manage the rehabilitation program and optimize the treatment goals for individual patients.

Despite increasing literature were presented, the effectiveness of robotics for rehabilitation in upper limb motor poststroke rehabilitation remains inconclusive (Bertani et al., 2017; Veerbeek et al., 2017; Ferreira et al., 2018; Mehrholz et al., 2018; Chien et al., 2020). Robotic therapy adjunct to standard-intensity conventional therapy was more beneficial than standard intensity conventional therapy alone (Bertani et al., 2017; Veerbeek et al., 2017; Ferreira et al., 2018; Mehrholz et al., 2018). However, the meta-analysis also suggested that under similar training intensity, the improvement of upper limb function was comparable between robotic therapy and conventional therapy for stroke survivors (Veerbeek et al., 2017; Ferreira et al., 2018; Chien et al., 2020). It should be noted that those meta-analysis results derived in the aggregate of the general stroke population may not provide the best evidence of practice for stroke survivors with different levels of impairments (Winstein et al., 2016). Recent robotic rehabilitation studies reported that chronic stroke survivors with moderate deficits achieved greater improvement in motor function from robot-assisted upper limb training than those with mild motor deficits (Hsieh et al., 2012; Takahashi et al., 2016; Takebayashi et al., 2020). Therefore, stratification of stroke participants based on the impairment level is important in terms of estimating the recovery pattern and prognostication of outcomes (Veerbeek et al., 2017). Moreover, research in robotic training in early stroke rehabilitation is still scarce, particularly for the elbow joint. Elbow extension/flexion is essential for upper limb function such as reaching and grasping, while the elbow joint is also the most common and long-lasting affected post-stroke (Roby-Brami et al., 2003). To our knowledge, there is a lack of available exoskeleton robots targeting the elbow joint for in-bed stroke rehabilitation. Most of the existing exoskeleton robots are complex and expensive (Veerbeek et al., 2017) that limits their application in the in-patient clinical setting. Meanwhile, an end-factor controlled robot may not be suitable for subacute patients with moderate and severe upper limb control. As an alternative, a portable exoskeleton elbow robot would be beneficial for in-patient upper limb rehabilitation. Motivated by the unmet need, we have developed a wearable elbow robot that can provide both passive stretching and active game-based training. The active and passive robotic training modalities have been suggested to be feasible and effective in ankle rehabilitation post-stroke (Ren et al., 2017).

The purpose of the present study was to conduct in-patient rehabilitation training on subacute stroke survivors with moderate and severe upper limb motor impairment using a wearable elbow rehabilitation robot. We aimed to: (1) evaluate the feasibility and effectiveness of a wearable elbow robotic device in subacute stroke in-bed training; and (2) investigate the active motor recovery patterns of stroke survivors with severe and moderate levels of motor impairments. It was assumed that: (1) a 4-week in-bed robot-guided training would improve elbow biomechanical properties and motor function; (2) patients with different motor impairment levels at the baseline would have different motor recovery patterns.

## Materials and Methods

### Participants

Patients with early subacute (7 days to 3 months post-stroke) and late subacute (3–6 months) stroke were enrolled in this study (Bernhardt et al., 2017). Inclusion criteria were: (1) age of 18–79 years old; (2) first episode of stroke verified through computed tomography or magnetic resonance imaging; (3) within 6 months post-stroke with impaired elbow motor function (grading of hemiplegic elbow joint Medical Research Council <4), (4) absence of any medical contraindication to exercise; (5) no gross visuospatial or visual field deficits which interfered with feedback training using a computer monitor; (6) the ability to understand and follow oral instructions (follow direction by order obey ≥1 step); and (7) medically stable.

Exclusion criteria were: (1) traumatic brain injury; (2) subarachnoid hemorrhage or lacunar infarct without apparent hemiplegia or hemiparesis; (3) previous upper limb amputation; (4) previous musculoskeletal problems on the impaired side including severe arthropathy, arthritis, or complicated orthopedic surgery on either side; (5) other degenerative neurologic problems such as Parkinsonism, Alzheimer’s dementia, or known other dementia; (6) skin lesion, acute infection on application site of the robotic arm; and (7) multiple stroke with neurological sequelae. The study was carried out in conformity with the Declaration of Helsinki; all patients gave their informed consent to participate in the study, which had been approved by the local scientific and ethics committees.

### The Wearable Elbow Robot Device

**Figure 1:**
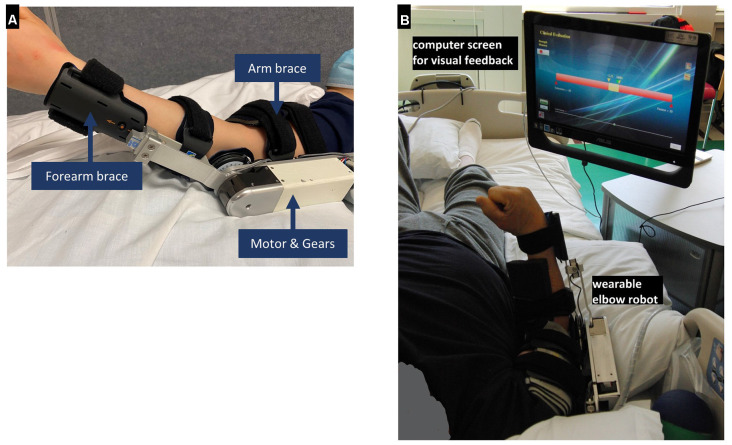
**(A)** The wearable elbow robot used for training. **(B)** Clinical in-bed setup using the wearable rehabilitation robot. It is worn by a patient on the elbow for controlled passive stretching and active movement training with robotic assistance or resistance or with real-time feedback during training. A force sensor was used to detect the elbow flexion and extension torque.

A wearable elbow robot (Rehabtek LLC, Linthicum Heights, MD, USA) with audiovisual feedback was used for the in-bed elbow movement training (Figure 1A). The exoskeleton robot included the upper arm and forearm braces, a servomotor (EC-4 poles, 120W, Maxon Powermax, Sachseln, Switzerland) with a gearhead (GP32C, ratio 86:1, Maxon Powermax, Sachseln, Switzerland) and a bevel gear with a ratio of 3:1. The driving linkage was connected to the forearm brace through a force sensor (MLP-50, nonlinearity 0.05 lb, Transducer Techniques, Temecula, CA, USA) to determine the elbow joint torque. The output axis of the bevel gear was aligned with the elbow flexion axis and flexed/extended the elbow joint through the force sensor and forearm brace.

The wearable robot was designed to provide passive stretching, game-based active movement training with the assist-as-needed scheme, and evaluation of biomechanical properties, including muscle strength and elbow range of motion. The wearable robot was interfaced through a touchscreen computer for display and user interface (Figure 1B). The user interface allowed adjustment of the applied torque value, movement velocity, and difficulty levels of the active movement games, such as assistance or resistance level (i.e., assisted-as-needed scheme) according to the patient’s ability.

### Elbow Robot Training Set-Up and Procedures

Patients lay supine in bed and wore the wearable robot on the paretic arm, with the shoulder at about 30-degree flexion and 15-degree abduction. The elbow robot was carefully mounted on the affected elbow with the brace adjusted to align the elbow flexion axis along with the wearable robot output axis (Figure 1A). The computer monitor was put in front of the patient with height and angle adjusted for proper viewing (Figure 1B). To determine a safe range of robot movement, the operator manually moved the elbow to its end of flexion/extension within the tolerance of patients.

**Figure 2:**
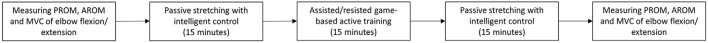
Elbow robot training procedure.

The training procedures are shown in Figure 2. Each training session typically consisted of passive intelligent stretching of the elbow (15 min), active-assisted and/or resisted movement training through movement gameplay (15 min), and passive intelligent stretching for cool down (15 min). Elbow active range of motion (ROM) and maximum isometric voluntary contraction (MVC) of elbow flexors and extensors were measured before and after each session of training. The training protocol would be adjusted individually to accommodate the condition of patients with severe hemiplegia including more passive stretching and less active movement training while therapy intensity was maintained ~150 repetitions of elbow flexion/extension passively or actively.

During the passive intelligent stretching, the forearm was passively moved by the robot in the sagittal plane at 30–40°/s. As the resistance may increase near the extreme positions of the elbow joint, the robot gradually slowed down to stretch the muscle-tendon complex slowly and safely (Ren et al., 2017). Once a predefined peak resistance torque (e.g., 5 Nm) was reached, the elbow joint was held at the extreme position for 10 s to allow soft tissue stress relaxation (Ren et al., 2017). During stretching, the patient was instructed to relax, feel the stretch but not to react to it (Ren et al., 2017). If the patient reacted to the stretching with high resistance, the robot would stop if a resistance torque limit was reached or reverse the direction of movement if resistance torque was beyond the limit (Ren et al., 2017).

Two types of active movement training were completed by the participants by voluntarily flexing and extending their elbow to play various movement games under real-time feedback, in which the robot could provide assistance after the patients tried but could not finish the movement task, or the robot provided resistance to challenge the patients if they could move the elbow to the target positions in the gameplay. Robot assistance during patient’s active movement training helped the patients reach the target and kept them engaged, while robot resistance continued challenging the patients to generate muscle strength (Waldman et al., 2013; Ren et al., 2017). The choice of assistive or resistive type of active movement training was dependent on the patient’s severity of elbow impairment. For patients at the early stage of recovery with little elbow movement capability, the wearable rehabilitation robot constrained the joint at an isometric condition and the patient’s potential re-emerging force-generation was detected sensitively by the wearable robot, and shown in real-time to the patient through visual feedback as a yellow bar on the computer monitor to guide the patient to generate the desired joint torque output (Figure 1B).

Participants received four sessions per week during their about 4-week hospital stay, for a total of about 15 sessions. The training protocol would be adjusted individually to accommodate the condition of patients with severe hemiplegia including more passive stretching, and less active movement training while therapy intensity was maintained ~150 repetitions of elbow flexion/extension passively or actively. In addition to the robotic training, all the patients also received their regular inpatient rehabilitation including physical therapy and occupational therapy.

### Outcome Evaluation

Biomechanical outcome measures were conducted immediately before and after the robotic training, including muscle strength measured as a maximum isometric voluntary contraction (MVC) of elbow flexors and extensors. During the measurement, the wearable robot was locked at the 90° elbow flexion and the participant was encouraged to extent and flex his or her elbow maximally. Each measure was done three times with a rest break of 30–60 s to minimize fatigue. The measured data were saved in the robot computer and the averaged value of the three assessments was taken as the corresponding outcome measure.

Clinical outcome measures, including upper limb motor recovery, muscle strength, and spasticity, were made on all the participants before and after all the robot-aided training (i.e., about 4 weeks from baseline) and 4 weeks after the completion of the training.

Fugl-Meyer Assessment of upper extremity (FMA-UE) was used to evaluate motor recovery (maximum score of 66) with a higher score indicating better motor recovery (Gladstone et al., 2002). The cut-off score for severe, moderate, and mild upper limb motor impairment is suggested to be 26 and 53 (Woodbury et al., 2013). Muscle strength was also evaluated using Motricity Index for the upper extremities (MI). As a valid muscle strength evaluation scale of stroke recovery in the first 6 months post-stroke, MI assessed the muscle strength of shoulder abduction, elbow flexion, and pinch grip. The total score ranges from 0 to 99, with a higher score corresponding to better muscle strength (Bohannon, 1999).

Spasticity of the elbow flexors and extensors was evaluated using the Modified Ashworth Scale (MAS). Considering the 1+ score is not ordinal, the scores of 0, 1, 1+, 2, 3, and 4 were adjusted to 0–5 ordinal scores in further analysis, with a higher value indicating more severe muscle spasticity (Pandyan et al., 1999a; Ansari et al., 2008).

### Data Analysis

The normality of variables was checked using the Shapiro-Wilk test. Due to the small sample size, non-parametric analysis was used for the study. For all outcome variables, the group mean and standard deviation or median and inter-quartile range (IQR) at pre-and post-training, and follow-up were calculated. For the clinical outcome measures, the Friedman test was used to test whether the change between pre-, post-, and follow-up was statistically significant. Paired comparisons using the Wilcoxon signed-rank test were made between pre-and post-training conditions and between pre-and follow-up with Bonferroni adjustment. All statistical analyses were performed using the SPSS statistical software (Version 26, IBM, Armonk, NY, USA). The statistical significance was set at *p* < 0.05.

Furthermore, for MVC of elbow flexors and extensors, measured before and after each of the treatment sessions, improvement curves over sessions based on a logarithmic fitting equation as below (Kwakkel et al., 2006; Langhorne et al., 2011; Chen et al., 2018):

y=a×ln(x)+b

where *x* is the number of the training session, *y* is the MVC value, *a* denotes the rate of improvement and *b* indicates the initial muscle strength of the patient. The coefficient of determination (*R*^2^) was calculated to assess the goodness of fit.

## Results

### Participants

Eleven patients (mean age ± SD: 50.7 ± 10.6 years) with moderate to severe upper limb motor impairment [FMA-UE, median (IQR): 28 (14–45)] at subacute stroke stage (post-stroke duration: 2.6 ± 1.9 months) completed the training during their hospital stay of 28 days on average (range: 24–30 days). Table 1 summarizes the characteristics of each participant at the baseline. Three and eight patients were with severe and moderate upper limb motor impairment, respectively, at the baseline.

**Table 1:** Characteristics of the patients at the baseline^a^.

Patient	Sex	Age (years)	Stroke duration (months)	Stroke type	Lesion area	Hemiplegic side	Dominant hand	FMA-UE	MAS of flexors	MAS of extensors	Motricity index
1	Female	62	3	Infract	Temporal lobe	Left	Right	45	1	0	62.5
2	Male	63	2	Hemorrhage	Frontal lobe, cerebellum	Right	Right	14	1	0	25.5
3	Female	57	2	Hemorrhage	Thalamus	Right	Right	49	0	0	64
4	Female	39	5	Hemorrhage	Temporal lobe	Right	Right	37	0	0	65
5	Male	54	3	Hemorrhage	Brain stem	Right	Right	28	1	0	42.5
6	Male	44	6	Infract	Frontal lobe, basal ganglia	Left	Right	7	0	0	63.5
7	Female	52	1	Hemorrhage	Basal ganglia	Right	Right	30	1	0	30
8	Female	56	1	Infract	Thalamus, basal ganglia	Left	Right	26	0	0	52
9	Female	71	3	Hemorrhage	Thalamus, basal ganglia	Left	Right	28	0	1	57.5
10	Male	45	2	Infract	Frontal lobe, basal ganglia	Right	Right	52	0	1	62
11	Male	37	1	Infract	Frontal, temporal, and parietal lobe	Left	Right	5	0	0	N/A
Mean	Male: *n* = 7; Female: *n* = 4	50.7	2.6	Infarct: *n* = 5; Hemorrhage: *n* = 6		Left: *n* = 5; Right: *n* = 6	Right: *n* = 11	28^b^	0^b^	0^b^	52.5
SD		10.6	1.9					14–45^b^	0–1^b^	0–0^b^	14.0

*^a^FMA-UE, Fugl-Meyer Assessment-Upper Extremities; MAS, Modified Ashworth Scale; N/A, not applicable to conduct*. *^b^Median (inter-quartile range); SD, standard deviation*.

### Feasibility

We applied the robot training in accordance with their routine in-patient treatment schedule and there was no dropout in the patients. Mild skin compression due to robot fixation and muscle soreness were reported by six patients after the first session of training, but this symptom was relieved within 24 h after onset of the symptoms. In general, the in-bed elbow robot training was well-tolerated by the participants without other adverse events. Every participant was able to complete the 50-min training and reported satisfaction with passive stretching and occasionally mild fatigue following the active movement when asked by the researchers. Except one patient, who was discharged after 13-sessions of training, the other 10 patients completed 15 sessions of training. Due to the researcher applied assistance to patients during the muscle strength testing, the biomechanical measures for three patients were excluded from the analysis. Thus, in the following in-session and curve-fitting analysis, biomechanical data for eight patients over 13 sessions were included.

### Biomechanical Outcomes

#### Improvements After the 3–4 Week Training Program

**Figure 3:**
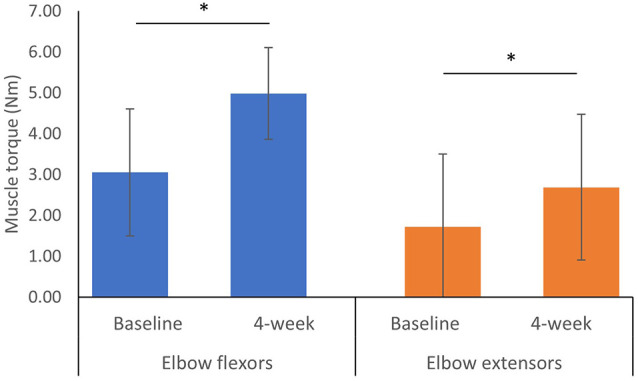
Maximum isometric voluntary contraction at baseline and 4-week after the training program^a^. ^a^Error bars represent standard deviation. *Indicates a significant difference between two measured time points from Wilcoxon Signed Ranks Test *p* < 0.05.

After 3–4 week of training during the hospital stay, the MVC of elbow flexors significantly increased by 1.93 Nm (95% CI: 0.93 to 2.93 Nm, *p* = 0.017) and the MVC of elbow extensors significantly increased by 0.68 Nm (95% CI: 0.05 to 1.98 Nm, *p* = 0.036; Figure 3).

#### In-Session Changes Over the 4-Week Training Program

**Figure 4:**
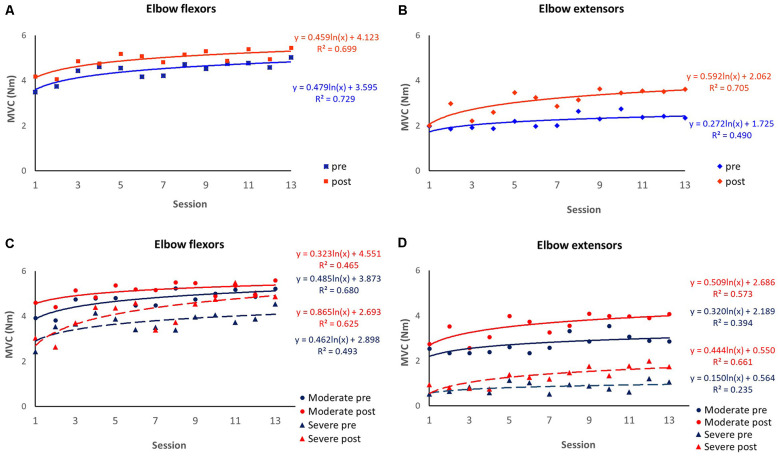
Session-by-session pre-and post-session maximum isometric voluntary contraction (MVC). Improvement curves across the 13 sessions are fitted with logarithmic function *y* = *a*×ln(*x*) + *b*, where *x* is the number of the training session, *y* is the MVC value, a denotes the rate of improvement and *b* indicates the initial performance capability level of the patients. The coefficient of determination (*R*^2^) was calculated to assess the goodness of fit. **(A)** MVC of elbow flexors over eight patients. **(B)** MVC of elbow extensors over eight patients. **(C)** MVC of elbow flexors over patients with severe motor impairment (*n* = 2) and moderate motor impairment (*n* = 6). **(D)** MVC of elbow extensors over patients with severe motor impairment (*n* = 2) and moderate motor impairment (*n* = 6).

Each session of robot-guided training-induced changes in the MVC of elbow flexors and extensors, as indicated by the pre-and post-session dot plots over 13 sessions are shown in Figure 3. These recovery curves showed overall improvement in the patients’ motor control ability over 13 training sessions, as well as the improved performance due to each training session, as shown by the differences between the pre-and post-session improvement curves (the blue and red curves respectively). We plot the overall change for eight patients with biomechanical measures, and further plot the recovery curve for patients with moderate upper limb motor impairment (FMA-UE motor >26, *n* = 5) and severe motor impairment (FMA-UE motor ≤26, *n* = 2). Overall, the improvement rate derived from the post-session measures was larger than the pre-session measures for both elbow flexion MVC (Figure 4A) and extension MVC (Figure 4B), which was related to the improvement induced by each training session. For the elbow flexors (Figure 4C), overall, patients with severe motor impairment had a lower initial performance value (pre: *b* = 2.898; post: *b* = 2.693) than those with moderate motor impairment (pre: *b* = 3.873; post: *b* = 4.551), and the post-session improvement rate was larger in the severe motor impairment group (*a* = 0.865) compared to the moderate motor impairment group (*a* = 0.323).

**Figure 5:**
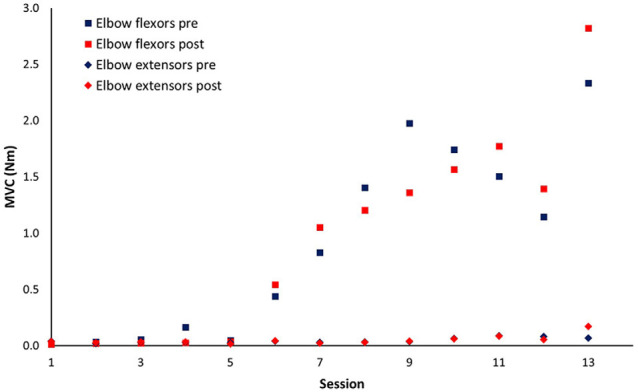
Session-by-session pre-and post-session maximum isometric voluntary contraction (MVC) for patient No.11. This patient started to develop elbow flexors voluntary contraction after five sessions of training.

Patient No. 11 with very severe motor impairment (FMA-UE = 5) at baseline was unable to generate active elbow movement in the first five sessions of training. His recovery curve was discontinuous due to the important zero to non-zero motor output change and was modeled separately (Figure 5).

### Clinical Outcomes

The clinical measures were conducted before and after 15 sessions of training (except for one patient discharged after 13 sessions of training), and 4 weeks after the termination of training. After 4 weeks of training, significant improvements were observed in FMA-UE (*p* = 0.003) and Motricity Index (*p* = 0.005; Table 2), and the improvements sustained at the follow-up (*p* < 0.05). However, no significant differences were observed between the post-training and 4-week follow-up. In addition, subscale value of FMA are presented in Table 2. Generally, participants showed improvement in movement, while no significant change was observed in coordination. There was no significant change of muscle spasticity measured by MAS between the baseline, post-training, and follow-up sessions.

**Table 2:** Clinical measures at baseline, post-training and follow-up^a^.

	Baseline		Post-training		Follow-up		*p*-value	
	Median	IQR	Median	IQR	Median	IQR	Pre-post	Pre-follow-up
FMA-UE	28	14–37	42	19–51	51	36–53	0.003*	0.005*
Shoulder/elbow	17	14–26	28	19–30	29	18–32	0.008*	0.008*
Wrist	5	2–6	7	4–9	7	6–9	0.007*	0.005*
Hand	6	2–8	9	4–12	11	7–12	0.005*	0.005*
Coordination	0	0–2	2	0–4	2	2–5	0.068	0.041*
Motricity index	60	40–64	70	60–71	70	60–73	0.005*	0.005*
MAS elbow flexors	0	0–1	0	1–1	0	0.5–1	0.083	0.157
MAS elbow extensors	0	0–0	0	0–1	0	0–1	0.564	0.414

^a^FMA-UE, Fugl-Meyer Assessment-Upper Extremities; IQR, inter-quartile range; MAS, Modified Ashworth Scale. *Statistically significant difference using Wilcoxon signed rank test, *p* < 0.05.

## Discussion

Our findings suggest that the in-bed wearable elbow robotic rehabilitation training is feasible in early and late subacute stroke survivors with moderate to severe upper limb motor impairment. Over the 4 weeks of training, participants made consistent improvements on elbow biomechanical and clinical outcomes, while the recovery curve showed that patients with severe motor impairment may have a greater rate of improvement compared to those with moderate motor impairment. The current findings address the valuable application of in-bed rehabilitation robotic training for subacute stroke survivors with its advantages in quantifying and monitoring the motor recovery and delivery of the individualized intervention, though our results must be interpreted with obvious caution given the absence of a control group.

The wearable elbow robot training combined with both passive and active movement has several unique features. First, the intelligent control algorithm provides a forceful while safe stretching to the upper limb muscles, which can prevent muscle contracture and joint stiffness (Ren et al., 2017; Salazar et al., 2019). Also, the strong stretching may enhance somatosensory input that helps drive neural reorganization (Behm et al., 2016). Second, an assisted-as-needed scheme was applied in the active movement training, which would be particularly useful for the subacute stroke survivors with moderate to severe motor impairment that has limited capability to generate active movement. If the patient could generate active arm movement, the robot would provide resistance that further enhances the movement control; if the patient could initiate movement but with difficulty to complete the required movement, the robot would help the patient to finish the rest of the movement; or if the patient was unable to generate active movement, the robot would assist the patient to passively move the arm to the desired position. Indeed, applying passive movement before clinical recovery has been proven that can elicit cortical activation patterns that may be critical for the restoration of motor function (Matteis et al., 2003). The robotic assistance would progressively reduce through the training that further promotes motor learning (Winstein et al., 2016). Third, the robot with a highly sensitive force sensor can discern the emergence of the subtle change of tiny movement. For patients, this subtle movement change was further augmented and displayed on the computer screen to provide real-time visual feedback that motivates and guides the patient to improve joint torque generation (Ren et al., 2017). For clinicians, the detection by the robot could provide essential information to optimize the treatment goal and assist rehabilitation plan. In Figure 5, the patient was unable to generate active muscle strength in elbow flexors until after five sessions of training, and this slight change was successfully detected by the robot.

Utilizing the robot, immediate biomechanical measures can be made before and after each training session, and further plot a recovery curve of the biomechanical outcome change (Figure 4). Though we can observe fluctuation of performance that may occur between sessions, overall, the patients demonstrated an improving trend over the training sessions. However, fluctuation patterns may imply that for longitudinal intervention trials, the multiple-session assessment may provide a better estimation of participants’ condition than a single-session assessment. Furthermore, consistent with previous clinical trials (Takahashi et al., 2016; Takebayashi et al., 2020), our stratified recovery curve informed that patients with severe motor impairment were likely to benefit more from robotic training compared to those with moderate motor impairment. We believe this recovery could provide important information to guide the clinical application of robot training (Veerbeek et al., 2017). First, the recovery curve can assist clinicians to estimate the recovery pattern of the patients, thus optimizing the treatment plan; second, with a larger sample size to plot the recovery curve, we may be able to estimate the minimal training sessions to achieve the desired outcome, which may assist Medicare policymaking.

A limitation of the present study is the lack of a control group to isolate the effect of spontaneous motor recovery. However, we applied the clinical measures including FMA-UE and Motricity Index (MI) for upper limb muscle strength at baseline, immediately after the intervention program, and 1-month after the termination of the program, which could serve as a self-control comparison (Table 2). Immediately after the training program, the median score of FMA-UE significantly increased to 14, which is larger than 9, the minimal clinically important difference (MCID) value of FMA-UE for subacute poststroke patients (Narayan Arya et al., 2011). After a 1-month follow-up, there were significant changes in the FMA-UE and MI. Collectively, the outcome of clinical measures could further support the effectiveness of in-bed wearable training in the improvement of upper limb motor limb function and minimize the confounding of spontaneous recovery.

There are limitations to the study. First, patients in the study also received routine in-patient rehabilitation, which could contribute to their improvement. However, this study demonstrated the feasibility to incorporate the in-bed robot training with routine inpatient rehabilitation training, which may not focus on sensorimotor rehabilitation. Anecdotally, the patients enjoyed the robot training and were highly motivated. However, we did not have a treatment satisfaction rating using the Likert system to evaluate patients’ responses, which should be adopted in future studies. Second, patients in the present study had mild or absent elbow spasticity which may limit the generalizability of the study. The mild or absent elbow spasticity would be due to most of the patients being at the early subacute stage (7 days to 3 months post-stroke; Bernhardt et al., 2017) that spasticity had not been developed yet (Wissel et al., 2013). In fact, one patient 1-month post-stroke with zero MAS at the elbow showed wrist spasticity of two at the follow-up and two other early subacute patients showed an increase of MAS from zero at the baseline to one at post-training. Also, the forceful passive elbow stretching by the robot might help control spasticity (Ren et al., 2017). Nonetheless, appropriate quantification of spasticity is important. MAS may not be a valid and ordinal level measure of muscle spasticity (Pandyan et al., 1999b). Future studies can consider using the modified Modified Ashworth Scale, which has been suggested to be better inter-rater and intra-session reliability than the MAS to measure spasticity (Ansari et al., 2006, 2009). Also, Brunnstrom recovery stages (BRS) can be used to evaluate the changes of muscle tone, synergistic movements, and active isolated movement (Naghdi et al., 2010). Third, only a small number of in-patients participated in the study. The goodness-of-fit value for curve fitting was thus relatively low. In the future, a strictly designed randomized control trial with a large sample size with different motor impairment levels is needed.

In conclusion, the above study demonstrated the feasibility of using in-bed wearable elbow robot-aided rehabilitation training in subacute stroke survivors with moderate to severe upper limb motor impairment. Furthermore, robotic therapy may result in significant improvement across biomechanical and clinical measures. The recovery curve generated from the robot biomechanical measures could provide useful information to guide the clinical applications of robot-aided training. Patients with severe motor impairment may benefit more from the robot training compared to those with less severe impairment.

## Data Availability Statement

The raw data supporting the conclusions of this article will be made available by the authors, without undue reservation.

## Ethics Statement

The studies involving human participants were reviewed and approved by the ethics committee of Presbyterian Medical Center, Jeonbuk, South Korea. The patients/participants provided their written informed consent to participate in this study.

## Author Contributions

All authors listed have made a substantial, direct and intellectual contribution to the work, and approved it for publication.

## Conflict of Interest

L-QZ has an ownership in Rehabtek LLC, which received U.S. federal fundings in developing the rehabilitation robot used in this study. The remaining authors declare that the research was conducted in the absence of any commercial or financial relationships that could be construed as a potential conflict of interest.

## References

[B1] Ansari N. N. Naghdi S. Arab T. K. Jalaie S. (2008). The interrater and intrarater reliability of the Modified Ashworth Scale in the assessment of muscle spasticity: limb and muscle group effect. NeuroRehabilitation 23, 231–237. 10.3233/nre-2008-23304 18560139

[B2] Ansari N. N. Naghdi S. Hasson S. Mousakhani A. Nouriyan A. Omidvar Z. (2009). Inter-rater reliability of the Modified Modified Ashworth Scale as a clinical tool in measurements of post-stroke elbow flexor spasticity. NeuroRehabilitation 24, 225–229. 10.3233/NRE-2009-0472 19458429

[B3] Ansari N. N. Naghdi S. Moammeri H. Jalaie S. (2006). Ashworth Scales are unreliable for the assessment of muscle spasticity. Physiother. Theory Pract. 22, 119–125. 10.1080/09593980600724188 16848350

[B4] Behm D. G. Blazevich A. J. Kay A. D. Mchugh M. (2016). Acute effects of muscle stretching on physical performance, range of motion and injury incidence in healthy active individuals: a systematic review. Appl. Physiol. Nutr. Metab. 41, 1–11. 10.1139/apnm-2015-0235 26642915

[B5] Bernhardt J. Dewey H. Thrift A. Donnan G. (2004). Inactive and alone: physical activity within the first 14 days of acute stroke unit care. Stroke 35, 1005–1009. 10.1161/01.STR.0000120727.40792.40 14988574

[B6] Bernhardt J. Hayward K. S. Kwakkel G. Ward N. S. Wolf S. L. Borschmann K.. (2017). Agreed definitions and a shared vision for new standards in stroke recovery research: the stroke recovery and rehabilitation roundtable taskforce. Int. J. Stroke 12, 444–450. 10.1177/1747493017711816 28697708

[B7] Bertani R. Melegari C. Maria C. Bramanti A. Bramanti P. Calabrò R. S. (2017). Effects of robot-assisted upper limb rehabilitation in stroke patients: a systematic review with meta-analysis. Neurol. Sci. 38, 1561–1569. 10.1007/s10072-017-2995-5 28540536

[B8] Bohannon R. W. (1999). Motricity index scores are valid indicators of paretic upper extremity strength following stroke. J. Physical Therapy Sci. 11, 59–61. 10.1589/jpts.11.59

[B9] Buma F. Kwakkel G. Ramsey N. (2013). Understanding upper limb recovery after stroke. Res. Neurol. Neurosci. 31, 707–722. 10.3233/RNN-130332 23963341

[B10] Chen K. Xiong B. Ren Y. Dvorkin A. Y. Gaebler-Spira D. Sisung C. E.. (2018). Ankle passive and active movement training in children with acute brain injury using a wearable robot. J. Rehabil. Med. 50, 30–36. 10.2340/16501977-2285 29104998

[B11] Chien W. T. Chong Y. Y. Tse M. K. Chien C. W. Cheng H. Y. (2020). Robot-assisted therapy for upper-limb rehabilitation in subacute stroke patients: a systematic review and meta-analysis. Brain Behav. 10:e01742. 10.1002/brb3.1742 32592282 PMC7428503

[B12] Ferreira F. M. R. M. Chaves M. E. A. Oliveira V. C. Van Petten A. M. V. N. Vimieiro C. B. S. (2018). Effectiveness of robot therapy on body function and structure in people with limited upper limb function: a systematic review and meta-analysis. PLoS One 13:e0200330. 10.1371/journal.pone.0200330 30001417 PMC6042733

[B13] French B. Thomas L. H. Coupe J. Mcmahon N. E. Connell L. Harrison J.. (2016). Repetitive task training for improving functional ability after stroke. Cochrane Database Syst. Rev. 4:CD006073. 10.1002/14651858.CD006073.pub2 27841442 PMC6464929

[B14] Gladstone D. J. Danells C. J. Black S. E. (2002). The fugl-meyer assessment of motor recovery after stroke: a critical review of its measurement properties. Neurorehabil. Neural Repair 16, 232–240. 10.1177/154596802401105171 12234086

[B15] Hsieh Y.-W. Wu C.-Y. Lin K.-C. Yao G. Wu K.-Y. Chang Y.-J. (2012). Dose-response relationship of robot-assisted stroke motor rehabilitation: the impact of initial motor status. Stroke 43, 2729–2734. 10.1161/STROKEAHA.112.658807 22895994

[B16] Johnson W. Onuma O. Owolabi M. Sachdev S. (2016). Stroke: a global response is needed. Bull. World Health Organ. 94:634. 10.2471/BLT.16.181636 27708464 PMC5034645

[B17] Krakauer J. W. (2006). Motor learning: its relevance to stroke recovery and neurorehabilitation. Curr. Opin. Neurol. 19, 84–90. 10.1097/01.wco.0000200544.29915.cc 16415682

[B18] Kundert R. Goldsmith J. Veerbeek J. M. Krakauer J. W. Luft A. R. (2019). What the proportional recovery rule is (and is not): methodological and statistical considerations. Neurorehabil. Neural Repair 33, 876–887. 10.1177/1545968319872996 31524062 PMC6854610

[B19] Kwakkel G. Kollen B. Twisk J. (2006). Impact of time on improvement of outcome after stroke. Stroke 37, 2348–2353. 10.1161/01.STR.0000238594.91938.1e 16931787

[B20] Kwakkel G. Kollen B. J. Van Der Grond J. Prevo A. J. (2003). Probability of regaining dexterity in the flaccid upper limb: impact of severity of paresis and time since onset in acute stroke. Stroke 34, 2181–2186. 10.1161/01.STR.0000087172.16305.CD 12907818

[B21] Lang C. E. Macdonald J. R. Reisman D. S. Boyd L. Kimberley T. J. Schindler-Ivens S. M.. (2009). Observation of amounts of movement practice provided during stroke rehabilitation. Arch. Phys. Med. Rehabil. 90, 1692–1698. 10.1016/j.apmr.2009.04.005 19801058 PMC3008558

[B22] Lang C. E. Wagner J. M. Edwards D. F. Dromerick A. W. (2007). Upper extremity use in people with hemiparesis in the first few weeks after stroke. J. Neurol. Phys. Therapy 31, 56–63. 10.1097/NPT.0b013e31806748bd 17558358

[B23] Langhorne P. Bernhardt J. Kwakkel G. (2011). Stroke rehabilitation. The Lancet 377, 1693–1702. 10.1016/S0140-6736(11)60325-5 21571152

[B24] Lee K. B. Lim S. H. Kim K. H. Kim K. J. Kim Y. R. Chang W. N.. (2015). Six-month functional recovery of stroke patients: a multi-time-point study. Int. J. Rehabil. Res. 38:173. 10.1097/MRR.0000000000000108 25603539 PMC4415968

[B25] Luker J. Lynch E. Bernhardsson S. Bennett L. Bernhardt J. (2015). Stroke survivors’ experiences of physical rehabilitation: a systematic review of qualitative studies. Arch. Phys. Med. Rehabil. 96, 1698–1708.e1610. 10.1016/j.apmr.2015.03.017 25847387

[B26] Matteis M. Vernieri F. Troisi E. Pasqualetti P. Tibuzzi F. Caltagirone C.. (2003). Early cerebral hemodynamic changes during passive movements and motor recovery after stroke. J. Neurol. 250, 810–817. 10.1007/s00415-003-1082-4 12883922

[B27] Mehrholz J. Pohl M. Platz T. Kugler J. Elsner B. (2018). Electromechanical and robot-assisted arm training for improving activities of daily living, arm function and arm muscle strength after stroke. Cochrane Database Syst. Rev. 9:CD006876. 10.1002/14651858.CD006876.pub5 30175845 PMC6513114

[B28] Mirbagheri M. M. Tsao C. Rymer W. Z. (2009). Natural history of neuromuscular properties after stroke: a longitudinal study. J. Neurol. Neurosurg Psychiatry 80, 1212–1217. 10.1136/jnnp.2008.155739 19060025

[B29] Naghdi S. Ansari N. N. Mansouri K. Hasson S. (2010). A neurophysiological and clinical study of Brunnstrom recovery stages in the upper limb following stroke. Brain Inj. 24, 1372–1378. 10.3109/02699052.2010.506860 20715900

[B31] Narayan Arya K. Verma R. Garg R. (2011). Estimating the minimal clinically important difference of an upper extremity recovery measure in subacute stroke patients. Top. Stroke Rehabil. 18, 599–610. 10.1310/tsr18s01-599 22120029

[B32] O’dwyer N. J. Ada L. Neilson P. D. (1996). Spasticity and muscle contracture following stroke. Brain 119, 1737–1749. 10.1093/brain/119.5.1737 8931594

[B33] Pandyan A. D. Johnson G. R. Price C. I. Curless R. H. Barnes M. P. Rodgers H.. (1999a). A review of the properties and limitations of the Ashworth and modified Ashworth Scales as measures of spasticity. Clin. Rehabil. 13, 373–383. 10.1191/026921599677595404 10498344

[B34] Pandyan A. D. Johnson G. R. Price C. I. Curless R. H. Barnes M. P. Rodgers H.. (1999b). A review of the properties and limitations of the Ashworth and modified Ashworth Scales as measures of spasticity. Clin. Rehabil. 13, 373–383. 10.1191/026921599677595404 10498344

[B35] Ren Y. Wu Y. Yang C. Xu T. Harvey R. L. Zhang L.. (2017). Developing a wearable ankle rehabilitation robotic device for in-bed acute stroke rehabilitation. IEEE Trans. Neural Syst. Rehabil. Eng. 25, 589–596. 10.1109/TNSRE.2016.2584003 27337720

[B36] Roby-Brami A. Feydy A. Combeaud M. Biryukova E. Bussel B. Levin M.. (2003). Motor compensation and recovery for reaching in stroke patients. Acta Neurol. Scand. 107, 369–381. 10.1034/j.1600-0404.2003.00021.x 12713530

[B37] Salazar A. P. Pinto C. Mossi J. V. R. Figueiro B. Lukrafka J. L. Pagnussat A. S.. (2019). Effectiveness of static stretching positioning on post-stroke upper-limb spasticity and mobility: systematic review with meta-analysis. Ann. Phys. Rehabil. Med. 62, 274–282. 10.1016/j.rehab.2018.11.004 30582986

[B38] Takahashi K. Domen K. Sakamoto T. Toshima M. Otaka Y. Seto M.. (2016). Efficacy of upper extremity robotic therapy in subacute poststroke hemiplegia: an exploratory randomized trial. Stroke 47, 1385–1388. 10.1161/STROKEAHA.115.012520 27006452

[B39] Takebayashi T. Takahashi K. Domen K. Hachisuka K. (2020). Impact of initial flexor synergy pattern scores on improving upper extremity function in stroke patients treated with adjunct robotic rehabilitation: a randomized clinical trial. Top. Stroke Rehabil. 27, 516–524. 10.1080/10749357.2020.1738660 32151236

[B40] Van Peppen R. P. Kwakkel G. Wood-Dauphinee S. Hendriks H. J. Van Der Wees P. J. Dekker J.. (2004). The impact of physical therapy on functional outcomes after stroke: what’s the evidence? Clin. Rehabil. 18, 833–862. 10.1191/0269215504cr843oa 15609840

[B41] Veerbeek J. M. Langbroek-Amersfoort A. C. Van Wegen E. E. Meskers C. G. Kwakkel G. (2017). Effects of robot-assisted therapy for the upper limb after stroke: a systematic review and meta-analysis. Neurorehabil. Neural Repair 31, 107–121. 10.1177/1545968316666957 27597165

[B42] Virani S. S. Alonso A. Benjamin E. J. Bittencourt M. S. Callaway C. W. Carson A. P.. (2020). Heart disease and stroke statistics—2020 update: a report from the American Heart Association. Circulation 141, E139–E596. 10.1161/CIR.0000000000000757 31992061

[B43] Volpe B. Krebs H. Hogan N. Edelstein L. Diels C. Aisen M.. (2000). A novel approach to stroke rehabilitation: robot-aided sensorimotor stimulation. Neurology 54, 1938–1944. 10.1212/wnl.54.10.1938 10822433

[B44] Waldman G. Yang C.-Y. Ren Y. Liu L. Guo X. Harvey R. L.. (2013). Effects of robot-guided passive stretching and active movement training of ankle and mobility impairments in stroke. NeuroRehabilitation 32, 625–634. 10.3233/NRE-130885 23648617

[B45] West T. Bernhardt J. (2012). Physical activity in hospitalised stroke patients. Stroke Res. Treat. 2012:813765. 10.1155/2012/813765 21966599 PMC3182066

[B46] Winstein C. J. Stein J. Arena R. Bates B. Cherney L. R. Cramer S. C.. (2016). Guidelines for adult stroke rehabilitation and recovery: a guideline for healthcare professionals from the American Heart Association/American Stroke Association. Stroke 47, e98–e169. 10.1161/STR.0000000000000098 27145936

[B47] Wissel J. Manack A. Brainin M. (2013). Toward an epidemiology of poststroke spasticity. Neurology 80, S13–S19. 10.1212/WNL.0b013e3182762448 23319481

[B48] Woodbury M. L. Velozo C. A. Richards L. G. Duncan P. W. (2013). Rasch analysis staging methodology to classify upper extremity movement impairment after stroke. Arch. Phys. Med. Rehabil. 94, 1527–1533. 10.1016/j.apmr.2013.03.007 23529144

